# Supplementary material for: Genome-Wide Association Study Adjusted for Occupational and Environmental Factors for Bladder Cancer Susceptibility
Source: Genes (Basel). 2022 Feb 28;13(3):448. doi: 10.3390/genes13030448 (PMC8950368; doi:10.3390/genes13030448)

Supplemental Figure S1: Results of a principle component analysis (A) Our samples and East Asian samples in IGSR. (B) Our samples and Chinese and Japanese samples in IGSR. The number of outliers from Japanese clusters was 8 and these samples were excluded.

(A) With East Asian samples

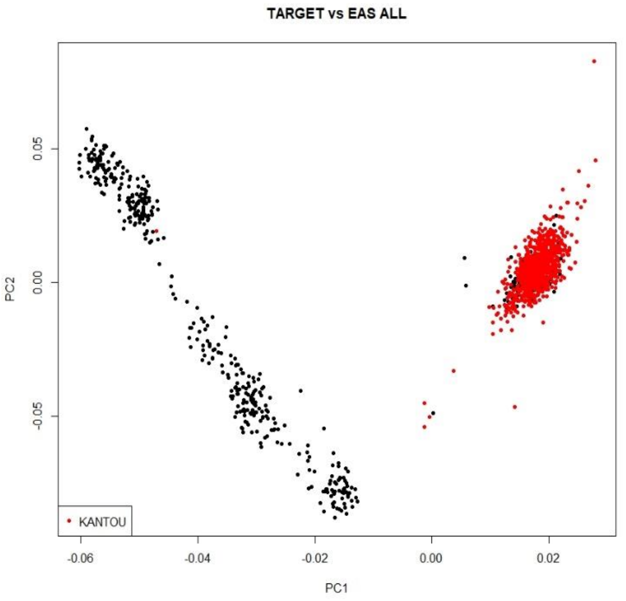

(B) With Chinese and Japanese samples

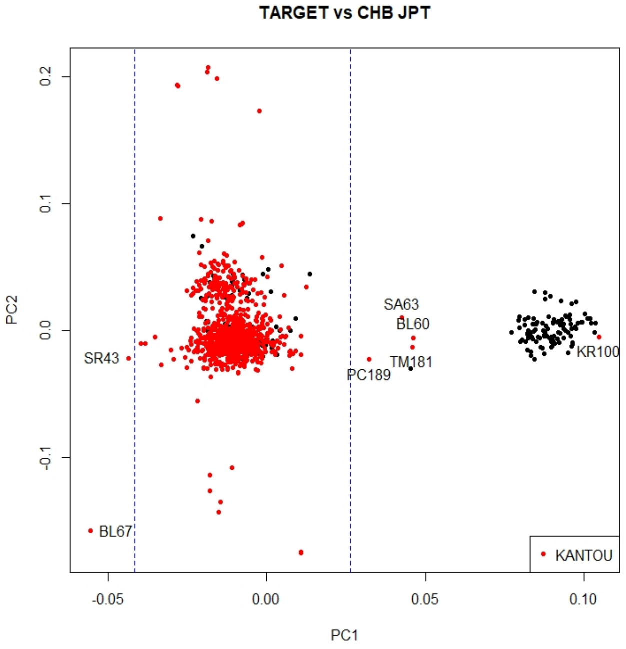

Supplement: Supplementary file 1 [file genes-13-00448-s001.zip › genes-1596190-supplementary/Supplements MDPI/Sup Figure S1.pdf]
