# Supplementary material for: Genome-Wide Association Study Adjusted for Occupational and Environmental Factors for Bladder Cancer Susceptibility
Source: Genes (Basel). 2022 Feb 28;13(3):448. doi: 10.3390/genes13030448 (PMC8950368; doi:10.3390/genes13030448)

Supplementary Figure S2: Regional plots of LINC00922 CDH5 (Upper) and LINC00473 PDE10A (Lower) regions. Added industrial/occupational factors were industrial classification divisions G and S for the upper panel and Zaitsu classifications for the lower.

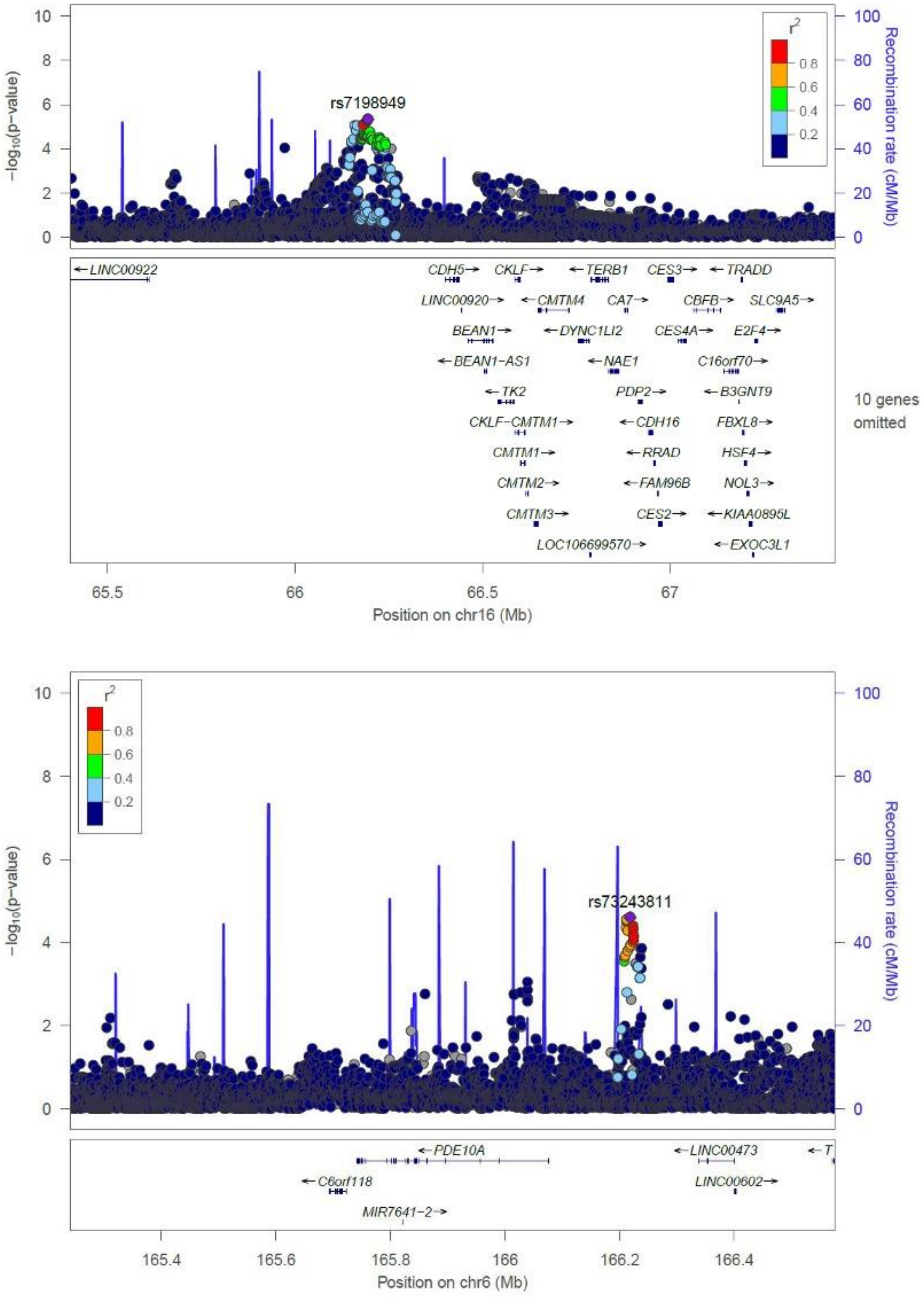

Supplement: Supplementary file 1 [file genes-13-00448-s001.zip › genes-1596190-supplementary/Supplements MDPI/Sup Figure S2.pdf]
