# Supplementary material for: Genome-Wide Association Study Adjusted for Occupational and Environmental Factors for Bladder Cancer Susceptibility
Source: Genes (Basel). 2022 Feb 28;13(3):448. doi: 10.3390/genes13030448 (PMC8950368; doi:10.3390/genes13030448)

Supplementary Figure S3: Regional plots of the WNT2B region. Added industrial/occupational factors were 1 variable with 20 levels for industrial classification divisions for the upper panel, industrial classification divisions G, L, and S for the middle, and selected industrial classification major groups in divisions D, E, and H for the lower.

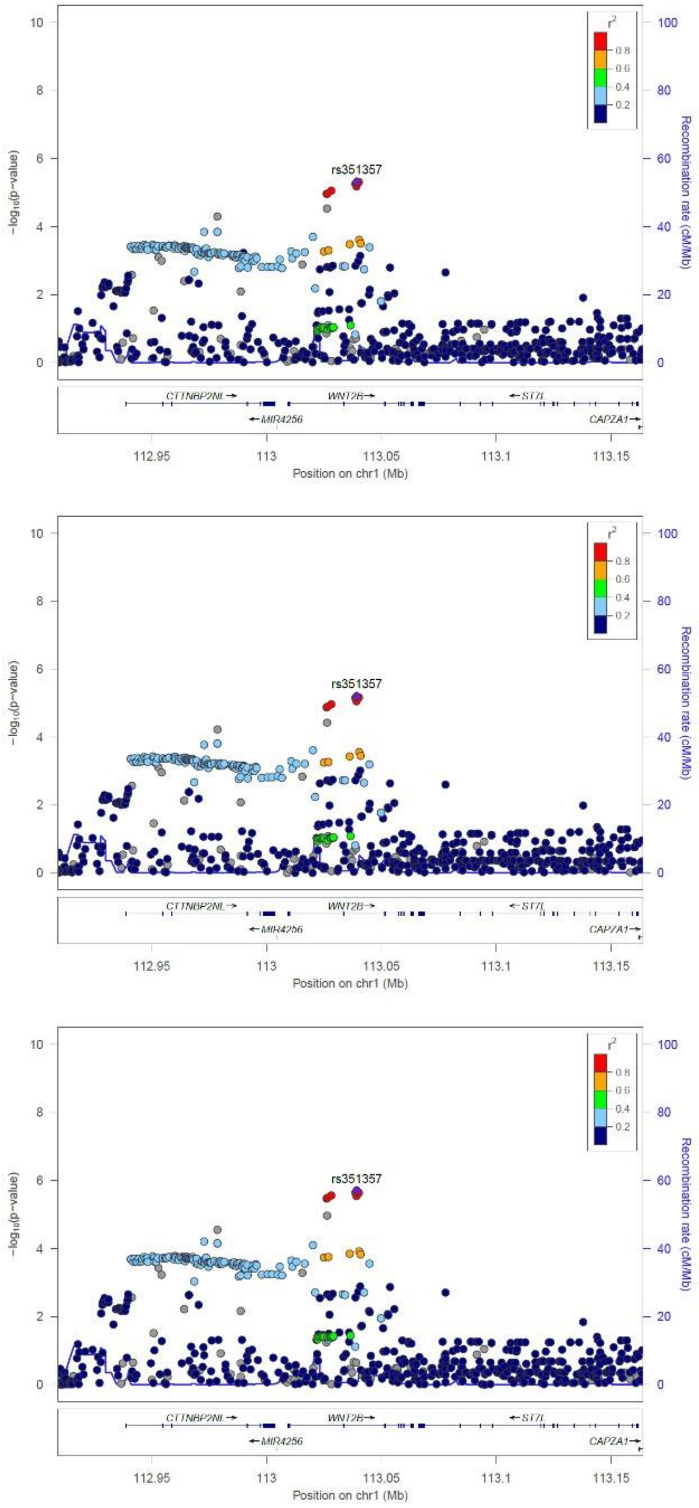

Supplement: Supplementary file 1 [file genes-13-00448-s001.zip › genes-1596190-supplementary/Supplements MDPI/Sup Figure S3.pdf]
