# Supplementary material for: Genome-Wide Association Study Adjusted for Occupational and Environmental Factors for Bladder Cancer Susceptibility
Source: Genes (Basel). 2022 Feb 28;13(3):448. doi: 10.3390/genes13030448 (PMC8950368; doi:10.3390/genes13030448)
Supplement: Supplementary file 1 [file genes-13-00448-s001.zip › genes-1596190-supplementary/Supplements MDPI/Sup Table S1.pdf]

Supplementary Table S1: SNPs susceptible to bladder cancer.

Variant and Risk Allele	*p*-Value	RAF	OR	CI	Mapped Gene	Location
rs9642880- T	7×10^{-12}	0.44	1.21	1.15-1.28	CASC11	8:127705823
rs798766- T	1×10^{-11}	0.19	1.24	1.17-1.32	TACC3	4:1732512
rs710521- A	6×10^{-8}	0.73	1.19	1.12-1.27	P3H2, TP63	3:189928144
rs2294008- T	2×10^{-10}	0.46	1.15	1.10-1.20	JRK, PSCA	8:142680513
rs710521- A	1×10^{-7}	0.73	1.19	1.12-1.27	P3H2, TP63	3:189928144
rs9642880- T	9×10^{-12}	0.45	1.22	1.15-1.29	CASC11	8:127705823
rs710521- A	2×10^{-10}	0.73	1.18	1.12-1.24	P3H2, TP63	3:189928144
rs1495741- ?	4×10^{-11}	0.80	1.15	1.10-1.20	NAT2	8:18415371
rs11892031- ?	1×10^{-7}	0.92	1.19	1.12-1.27	UGT1A8, UGT1A10	2:233656637
rs8102137- C	2×10^{-11}	0.33	1.13	1.09-1.17	CCNE1, C19orf12	19:29805946
rs9642880- T	2×10^{-18}	0.45	1.21	1.16-1.27	CASC11	8:127705823
rs798766- T	4×10^{-13}	0.19	1.20	1.14-1.26	TACC3	4:1732512
rs1014971- ?	8×10^{-12}	0.62	1.18	1.10-1.18	APOBEC3A, CBX6	22:38936618
rs2294008- T	4×10^{-11}	0.46	1.13	1.09-1.17	JRK, PSCA	8:142680513
rs401681- C	5×10^{-7}	0.54	1.11	1.07-1.16	CLPTM1L	5:1321972
GSTM1Del- ?	5×10^{-31}	0.51	1.47	1.38-1.57	-	
rs10094872- T	2×10^{-7}	0.41	1.26	-	CASC11	8:127707639
rs62185668- A	2×10^{-11}	0.24	1.19	1.13-1.26	FAT1P1, LINC02871	20:10981287
rs1711973- ?	3×10^{-6}	NR	-	-	FOXF2	6:1402302
rs2969540- ?	2 x 10-6	NR	-	-	RN7SKP280, HTR5A	7:155109147
rs3752645- ?	6×10^{-6}	NR	-	-	PRKAR2B	7:107142238
rs12216499- ?	1×10^{-6}	NR	-	-	RSPH3, RNU6-293P	6:158947492
rs1258767- ?	7×10^{-7}	NR	-	-	FMN1	15:32834253
rs17674580- T	8×10^{-11}	0.33	1.17	1.11-1.22	SLC14A1	18:45729946
rs7238033- ?	9×10^{-9}	-	1.20	1.13-1.28	SLC14A1	18:45737001
rs11543198- G	4×10^{-9}	0.78	1.41	1.26-1.59	CLK3	15:74619987
rs6104690- A	7×10^{-7}	0.56	1.12	1.08-1.18	LINC02871	20:11007451
rs907611- A	4×10^{-8}	0.31	1.15	1.09-1.21	LSP1	11:1852842
rs10936599- C	5×10^{-9}	0.76	1.18	1.11-1.23	MYNN	3:169774313
rs7747724- ?	1×10^{-6}	NR	1.11	1.06-1.16	CDKAL1	6:20751084
rs4510656- C	7×10^{-7}	0.55	1.12	1.08-1.18	CDKAL1	6:20766466
rs5003154- ?	1×10^{-6}	NR	1.11	1.06-1.16	PAG1	8:81074718
rs4907479- ?	3×10^{-6}	NR	1.13	1.07-1.18	MCF2L	13:113004794
rs11892031- A	1×10^{-7}	0.92	1.17	1.11-1.25	UGT1A8, UGT1A10	2:233656637
rs710521- A	2×10^{-11}	0.73	1.14	1.10-1.19	P3H2, TP63	3:189928144
rs798766- T	7×10^{-25}	0.19	1.22	1.18-1.27	TACC3	4:1732512
rs401681- C	4×10^{-11}	0.54	1.12	1.08-1.16	CLPTM1L	5:1321972
rs1495741- A	2×10^{-10}	0.80	1.14	1.09-1.18	NAT2	8:18415371
rs2294008- T	3×10^{-15}	0.46	1.13	1.10-1.16	AK6P2	12:38045401
rs9642880- T	4×10^{-38}	0.45	1.24	1.20-1.28	CASC11	8:127705823
rs10775480- T	6×10^{-8}	0.43	1.13	1.08-1.19	SLC14A1	18:45737317
rs8102137- C	1×10^{-11}	0.33	1.13	1.09-1.17	CCNE1, C19orf12	19:29805946
rs1014971- T	1×10^{-11}	0.62	1.13	1.09-1.17	APOBEC3A, CBX6	22:38936618
rs2042329- T	5×10^{-11}	0.10	1.40	1.27–1.55	CWC27	5:64771925
rs35356162- T	4×10^{-7}	0.00	4.33	2.463–7.619	UHRF1BP1	6:34834333
rs3736001- A	5×10^{-6}	0.10	1.27	-	PSCA, JRK	8:142681389
rs111249728- C	3×10^{-7}	NR	2.68	NR	IFNL3P1, SYCN	19:39230325
rs111812445- A	5×10^{-7}	0.02	1.82	NR	LINC01173	2:234754157
rs76088467- G	2×10^{-8}	0.02	1.56	NR	CASC15	6:21795556
rs10094872- T	3×10^{-11}	NR	1.23	NR	CASC11	8:127707639

rs56297045- A	2×10^{-7}	NR	1.23	NR	APOBEC3A	22:38954679
rs2736103- C	3×10^{-7}	NR	1.21	NR	TERT, MIR4457	5:1300286
rs2920281- T	2×10^{-8}	NR	1.19	NR	PSCA, JRK	8:142679026
rs13063162- A	5×10^{-7}	NR	1.16	NR	P3H2, TP63	3:189902464
rs10777753- A	7×10^{-8}	0.40	1.18	NR	CCDC38	12:95928824
rs11724531- A	8×10^{-12}	NR	1.28	NR	TACC3, FGFR3	4:1751697
rs180940944- ?	3×10^{-9}	NR	-		NBEA	13:35375956

modified from GWAS Catalog on Aug 31, 2021.
