# Supplementary material for: Genome-Wide Association Study Adjusted for Occupational and Environmental Factors for Bladder Cancer Susceptibility
Source: Genes (Basel). 2022 Feb 28;13(3):448. doi: 10.3390/genes13030448 (PMC8950368; doi:10.3390/genes13030448)
Supplement: Supplementary file 1 [file genes-13-00448-s001.zip › genes-1596190-supplementary/Supplements MDPI/Sup Table S2.pdf]

Supplementary Table S2: Japan Standard Industrial Classification (Rev. 13, October 2013), Divisions

Division	
A	Agriculture and Forestry
B	Fisheries
C	Mining and Quarrying Of Stone and Gravel
D	Construction
E	Manufacturing
F	Electricity, Gas, Heat Supply and Water
G	Information and Communications
H	Transport and Postal Services
I	Wholesale and Retail Trade
J	Finance and Insurance
K	Real Estate and Goods Rental and Leasing
L	Scientific Research, Professional and Technical Services
M	Accommodation, Eating and Drinking Services
N	Living-Related and Personal Services and Amusement Services
O	Education, Learning Support
P	Medical, Health Care and Welfare
Q	Compound Services
R	Services, N.E.C.
S	Government, Except Elsewhere Classified
T	Industries Unable to Classify
