# Supplementary material for: Genome-Wide Association Study Adjusted for Occupational and Environmental Factors for Bladder Cancer Susceptibility
Source: Genes (Basel). 2022 Feb 28;13(3):448. doi: 10.3390/genes13030448 (PMC8950368; doi:10.3390/genes13030448)
Supplement: Supplementary file 1 [file genes-13-00448-s001.zip › genes-1596190-supplementary/Supplements MDPI/Sup Table S3.pdf]

Supplementary Table S3: Japan Standard Occupational Classification (Rev. 5th, December 2009), Major groups

Major groups	
A	Administrative and managerial workers
B	Professional and engineering workers
C	Clerical workers
D	Sales workers
E	Service workers
F	Security workers
G	Agriculture, forestry and fishery workers
H	Manufacturing process workers
I	Transport and machine operation workers
J	Construction and mining workers
K	Carrying, cleaning, packaging, and related workers
L	Workers not classified by occupation
