# Supplementary material for: Genome-Wide Association Study Adjusted for Occupational and Environmental Factors for Bladder Cancer Susceptibility
Source: Genes (Basel). 2022 Feb 28;13(3):448. doi: 10.3390/genes13030448 (PMC8950368; doi:10.3390/genes13030448)
Supplement: Supplementary file 1 [file genes-13-00448-s001.zip › genes-1596190-supplementary/Supplements MDPI/Sup Table S4.pdf]

Supplementary Table S4: Incidence of malignant tumors other than urothelial cancer

	Prostate ca (%)	Renal cell ca	Testis tumor	Colorectal ca	Gastric ca	Lung ca	Laryngo-pharyngeal ca	Hematological malignancy	Mammary ca	Liver ca	Other malignancy	All
BladderCa_male (n=302)	2.3	0.3	0.0	3.6	2.6	1.7	0.7	0.7	0.0	1.0	1.0	13.9
Control_male (n=395)	46.6	11.1	1.3	3.5	3.8	2.3	1.0	1.5	0.0	0.3	0.8	72.2
BladderCa_female (n=50)	0.0	2.0	0.0	4.0	2.0	0.0	0.0	0.0	8.0	0.0	2.0	18.0
Control_female (n=39)	0.0	43.6	0.0	2.6	2.6	2.6	0.0	0.0	5.1	0.0	2.6	59.0
