# Supplementary material for: Genome-Wide Association Study Adjusted for Occupational and Environmental Factors for Bladder Cancer Susceptibility
Source: Genes (Basel). 2022 Feb 28;13(3):448. doi: 10.3390/genes13030448 (PMC8950368; doi:10.3390/genes13030448)
Supplement: Supplementary file 1 [file genes-13-00448-s001.zip › genes-1596190-supplementary/Supplements MDPI/Sup Table S5.pdf]

Supplementary Table S5: For 789 genotyped samples of 830 samples, 21 samples were excluded in which the sample call rate was <0.99, the proportion IBD was > 0.1875, and outliers from Japanese clusters identified by principal component analysis. In our study, we used 766 samples in GWAS.

| **Assessment** | | | |
|---|---|---|---|
| | Total | 830 | |
| | Successful experiment | 789 | |
| | | Extract | Exclude |
| | Call Rate ≧ 95% | 787 | 2 |
| | estimate IBD < 0.1875 | | 14 pair |
| | population structure | | 8 |
| Number of excluded samples | | | 21 |
| Number of samples for analysis | | 766 | |
