# Supplementary material for: Genome-Wide Association Study Adjusted for Occupational and Environmental Factors for Bladder Cancer Susceptibility
Source: Genes (Basel). 2022 Feb 28;13(3):448. doi: 10.3390/genes13030448 (PMC8950368; doi:10.3390/genes13030448)
Supplement: Supplementary file 1 [file genes-13-00448-s001.zip › genes-1596190-supplementary/Supplements MDPI/Sup Table S6.pdf]

Supplementary Table S6: We selected SNPs to be used for SNP imputation. The number of SNPs loaded on the chip was 659,184, and the number genotyped was 657,060. In addition, there were 641,043 SNPs with a definite chromosomal location, 395,708 SNPs with a call rate of 99% or higher, a p-value of 0.0001 for the Hardy-Weinberg law of equilibrium, and a minor allele frequency (MAF) of 1% or higher. SNP imputation was performed using 395,708 SNPs.

Assessment		Number of SNPs	
ALL		659,184	
Typing SNP		657,060	
		Extracted	Excluded
Mapping SNP		641,043	16,017
SNPQC			
	Call Rate ≧ 99%	566,144	74,899
	HWE p-value ≧ 0.001	565,007	1,137
	MAF ≧ 0.01	395,708	169,299
	Total		261,352
Number of selected SNPs for imputation		395,708	
