# Supplementary material for: Genome-Wide Association Study Adjusted for Occupational and Environmental Factors for Bladder Cancer Susceptibility
Source: Genes (Basel). 2022 Feb 28;13(3):448. doi: 10.3390/genes13030448 (PMC8950368; doi:10.3390/genes13030448)
Supplement: Supplementary file 1 [file genes-13-00448-s001.zip › genes-1596190-supplementary/Supplements MDPI/Sup Table S7.pdf]

Supplementary Table S7: The number of SNPs that was able to be imputed was 47,109,297. Of the 47,109,297 SNPs, 11,175,945 with an R-square value greater than 0.3 were used in the GWAS.

Number of Imputed SNPs	47,109,297	
	Extracted	Excluded
Number of SNPs satisfied Rsq≧0.3	11,175,945	35,933,352
Number of SNPs for analysis	11,175,945	0
